# Silencing of CASC8 inhibits non-small cell lung cancer cells function and promotes sensitivity to osimertinib via FOXM1

**DOI:** 10.7150/jca.47863

**Published:** 2021-01-01

**Authors:** Xizi Jiang, Jingqian Guan, Yitong XU, Hongjiu Ren, Jun Jiang, Muli Wudu, Qiongzi Wang, Hongbo SU, Yao Zhang, Bo Zhang, Zifang Zou, Yujiao Hu, Xiaodan SUN, Xueshan Qiu

**Affiliations:** 1 Department of Pathology, First Affiliated Hospital and College of Basic Medical Sciences, China Medical University, Shenyang, China.; 2 Department of Pathology, the First Bethune Hospital of Jilin University, Changchun, Jilin, China.; 3 Department of Pathology, Basic Medical Sciences, Xinjiang Medical University, Urumqi, China.

**Keywords:** Cancer susceptibility candidate 8, Long noncoding RNAs, Non-small Cell Lung, Forkhead Box Protein M1, Osimertinib

## Abstract

In a meta-analysis, the long noncoding RNA cancer susceptibility candidate 8 *(CASC8)* was found to be a cancer susceptibility gene closely related to lung cancer, but its functions in lung cancer are unknown. In the Cancer Genome Atlas database, the expression of *CASC8* was significantly higher in non-small cell lung cancer than in adjacent normal tissues, and high expression of *CASC8* was associated with poor prognosis in patients with lung adenocarcinoma. Silencing *CASC8* inhibited proliferation, migration, and invasion in non-small cell lung cancer cell lines. Silencing *CASC8* also promoted sensitivity to osimertinib through Forkhead box M1 (FOXM1). Therefore, this pathway can be exploited in patients with lung cancer resistant to targeted therapies. Our study revealed for the first time that silencing *CASC8* inhibited the proliferation, migration, and invasion of non-small cell lung cancer cells and promoted their sensitivity to osimertinib, suggesting that *CASC8* is closely related to the occurrence and development of non-small cell lung cancer. This may provide insight into mechanisms of treatment for non-small cell lung cancer.

## Introduction

Nearly 80% of lung cancers are non-small cell lung cancer (NSCLC) 1, and NSCLC is the leading cause of cancer-related deaths in the United States and China 2-4. Although surgery is the preferred method of treatment, only a portion of early lung cancers can be cured by surgical resection. Further, more than 60% of patients with lung cancer are diagnosed with locally advanced disease or have lymph node metastasis (stage III or IV), and surgery is not the primary treatment option. Patients with good physical condition usually receive chemotherapy first, and then choose whether surgery is needed according to their response 5. Although recent years have shown the discovery of many genetic drivers in NSCLC, such as epidermal growth factor receptor (*EGFR*) mutations, anaplastic lymphoma kinase fusion, and human telomerase reverse transcriptase overexpression, these drivers are at present the only therapeutic target genes for NSCLC 6-8. Studying the molecular mechanisms of NSCLC, identifying new therapeutic targets and improving the treatment of patients with lung cancer are necessary.

Long noncoding RNAs (lncRNAs) are RNAs longer than 200 nucleotides that lack a coding region 9. Although lncRNA is ubiquitously transcribed in humans and has a mature structure similar to that of mRNA, including its 5' capped architecture and 3' polyadenylation, lncRNA does not have the ability to be translated into protein 10,11. However, it has various regulatory functions, including cis-acting and trans-acting elements, transcriptional regulation, and signal transduction 12-14. LncRNAs can also act as a specific sponge of certain microRNAs (miRNAs), and adsorption of miRNAs inhibits their abilities 15-17. The lncRNA cancer susceptibility candidate 8 (*CASC8*) has been shown to be a tumor susceptibility gene 18, but its mechanism of action is not clear.

The aim of this study was to investigate how *CASC8* affects NSCLC through FOXM1 and examine the effect of *CASC8* on osimertinib drug sensitivity.

## Materials and Methods

### Patients and specimens

Tumors and corresponding normal tissues were collected from patients seen between 2015 and 2019 at the First Hospital Affiliated of China Medical University; the corresponding normal tissue samples were obtained from a location at least 5 cm away from the tumor tissue. The Chinese Medical University Institutional Review Committee approved the study, and all patients provided informed consent. Patients who participated in the study did not receive radiation therapy or chemotherapy before surgery. The clinical prognostic data involved in this study were provided by the Kaplan Meier-plotter database (https://kmplot.com/analysis/).

### Cell culture

The cell lines used in the study were all obtained from the Shanghai Cell Bank (Shanghai, China). Human bronchial epithelial (HBE) cells were cultured in high glucose Dulbecco's Modified Eagle Medium (DMEM); A549, H1299, and H460 cells were cultured in Roswell Park Memorial Institute (RPMI) 1640 media, these cell lines were all cultured in 10% qualified fetal bovine serum (FBS, FB15015; Clark Biosciences, Richmond, VA, USA); the H1975 and HCC827 cells were cultured in RPMI 1640 with 20% FBS.

### Plasmid construction and transfection

*CASC8* short hairpin RNA (113F078, CASC8-RNAi) and scramble small hairpin RNA-green fluorescent protein shRNA-GFP (negative control) were purchased from GENE (Shanghai, China). Transfection of shRNA was performed using Lipofectamine 3000 reagent (Invitrogen, Carlsbad, CA, USA) according to the manufacturer's instructions. shCASC8 or its negative control (NC) were transfected into selected cell lines, and the efficiency was tested after 24 hours. shRNA was also stably transfected into cell lines. Forty-eight hours after transient transfection, G418 (Thermo Fisher Scientific, Waltham, MA, USA) was added, and monoclonal stable-transfected cell lines were screened by limiting dilution. The cells were cultured in a 25T cell culture flask for subsequent experiments.

### Western blotting

Total protein was extracted with lysis buffer (P0013; Beyotime Biosciences, Shanghai, China); the volume depended on the number of cells collected. Appropriate amounts of protease inhibitor and phosphatase inhibitor were added to the lysis buffer (B14002 and B15002; Biotool, Shanghai, China). Thirty-five micrograms of each sample were separated by SDS-PAGE and the protein was transferred to a PVDF membrane (Millipore, Billerica, MA, USA). The membrane was blocked with 5% skim milk (232100; BD Co., Franklin Lakes, NJ, USA) for at least 2 hours, followed by gentle washing with Tween Tris buffered saline (TTBS) and overnight incubation at 4°C with the following primary antibodies: anti-Forkhead box M1 (FOXM1; 20459, 1:500 dilution, Cell Signaling Technology, Danvers, MA, USA), anti-E-cadherin (14472, 1:1000 dilution, Cell Signaling Technology), anti-Snail (2879, 1:1000 dilution, Cell Signaling Technology), anti-cyclin B1 (12231, 1:1000 dilution, Cell Signaling Technology), and anti-glyceraldehyde 3-phospate dehydrogenase (GAPDH) (TA319654, 1:1000 dilution, Origene). The next day, the membrane was incubated with peroxidase-conjugated secondary antibody for 2 hours at 37°C. Densitometry was quantitatively evaluated using ImageJ software.

### Quantitative real-time polymerase chain reaction (qPCR)

Ribonucleic acid (RNA) was extracted using TRIzol reagent (15596026, Thermo Fisher Scientific) and used to generate cDNA. After the complementary deoxynucleic acid (cDNA) was generated, an appropriate amount was taken for qPCR using the PrimeScript RT Master Mix (RR036A, TaKaRa, Japan). The reaction volume was 20 µL. The qPCR conditions were 95°C for 30 seconds and 40 cycles of 95°C for 5 seconds and 60°C for 30 seconds. A dissociation step was used to generate a dissolution profile and confirm whether the amplification was specific. Relative gene expression was calculated using the 2^-ΔΔCt^ method using GAPDH or U1 as controls. The primer sequences were as follows: *CASC8* forward, 5'-TGGCATGGACCAGGAGCACTAG-3′, and reverse, 5′-GGACTTGCCGGTAACCTAGATTGG-3′; U1 small nuclear 1 (*RNU1-1*) forward, 5'-ACCCCTGCGATTTCCCCAAA-3′, and reverse, 5′-CAGGGGAAAGCGCGAACG-3′;* GAPDH* forward, 5′-GGAGCGAGATCCCTCCAAAAT -3′, and reverse, 5′-GGCTGTTGTCATACTTCTCATGG-3′; and* FOXM1* forward, 5′-GATCTGCGAGATTTTGGTACAC-3′, and reverse, 5′-CTGCAGAAGAAAGAGGAGCTAT-3′.

### MTS proliferation assay

shCASC8 and NC cells were plated in 96-well culture plates at 3000 cells/well and cultured with cell strain-adapted FBS for 5 days in a sterile incubator at 37°C. A volume of 20 μL 3-(4,5-dimethylthiazol-2-yl)-5-(3-carboxymethoxyphenyl)-2-(4-sulfophenyl)-2H-tetrazole (MTS, G3580, Promega, Madison, Wisconsin, USA USA) was added to each well to test cell viability. After 1 hour incubation in a sterile incubator at 37°C in the absence of light, the absorbance at 490 nm was measured using a microplate reader to measure cell proliferation.

### Colony formation assay

A total of 500 cells/well of shCASC8 and NC cells were plated in 6-well culture plates. Four milliliters of RPMI 1640 were added to each well, and cells were incubated in a sterile incubator at 37°C for 10-14 days, at which point colonies were visible to the naked eye. For the colony formation experiment with osimertinib, 500 corresponding cells were plated in each well, the IC50 concentration of the drug was added for 48 hours, and cells were incubated in a sterile incubator at 37°C for 10-14 days. Cells were washed three times with PBS and then fixed with pre-cooled methanol. After fixation, the cells were again washed three times with PBS and then stained with crystal violet. The crystal violet was removed, and the cells were dried and photographed. Cell colonies were counted for statistical analysis.

### Invasion and migration analyses

Invasion assays were performed by adding 100 μL of Matrigel (1:9 dilution; BD Biosciences, San Jose, CA, USA) to an insert with a pore size of 8 μm (Costar, Washington, DC, USA). shCASC8 and NC cells were added to the transwell chamber and 600 μL RPMI 1640 with 20% FBS was added to the lower chamber. After 48 hours in a 37°C sterile incubator, cells in the upper chamber were removed with a cotton swab. Cells in the lower chamber were fixed with pre-cooled methanol, then stained with crystal violet. Cells were imaged and the number of cells that had passed through the transwell membrane was counted. Migration experiments were performed in the same fashion but without the use of Matrigel.

### Wound-healing assay

shCASC8 and NC cells were plated in a single layer in a 6-well culture plate. When the cells had reached 100% confluence, they were incubated with mitomycin (1758-9327, Inalco, Beijing, China) for 2 hours in a light-free environment, after which the monolayers were gently scraped with a 100-μL pipette tip under sterile conditions. After gently removing the scraped cells and washing the well with PBS, the scratch was imaged. Images were taken at the stated time points, and the distance was measured using PS software.

### Statistical analysis

All numerical data were analyzed by SPSS version 24.0 and GraphPad Prism 5. The χ^2^ test was used to assess the correlation between each indicator and *CASC8* expression. At least three independent replicates were performed in each experiment, and *P* < 0.05 was considered statistically significant.

## Results

### *CASC8* is upregulated in non-small cell lung cancer

**Figure 1:**
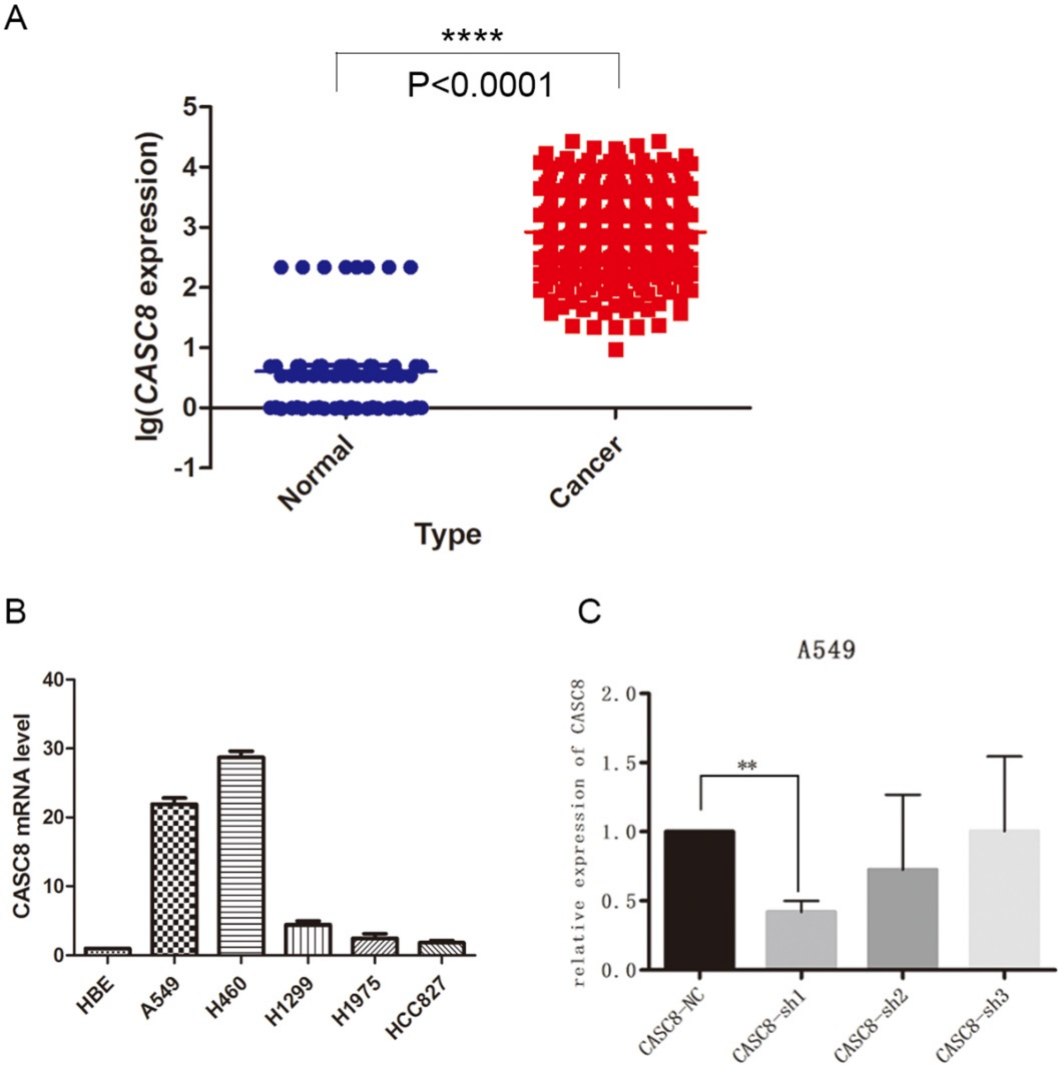
***CASC8* is upregulated in non-small cell lung cancer. A.** The expression of *CASC8* was analyzed in 535 lung cancer tissues and 59 normal lung tissues from TCGA database. The level of *CASC8* was higher in cancer tissues than in normal tissues. ***P < 0.0001. **B.** Expression of *CASC8* mRNA in the HBE (normal bronchial epithelial), A549 (NSCLC), H460 (NSCLC), H1299 (NSCLC), HCC827 (NSCLC), and H1975 (NSCLC) cell lines. **C.** Validation of *CASC8* knockdown cells. **P < 0.001. Abbreviations: CASC8: cancer susceptibility candidate 8; TCGA: The Cancer Genome Atlas; NSCLC: non-small cell lung cancer.

The lncRNA *CASC8* is a susceptibility factor for cancer, but its specific role in NSCLC is not well understood. In this study, we analyzed the expression of *CASC8* in NSCLC tissues. We downloaded expression data from The Cancer Genome Atlas (TCGA) database and analyzed *CASC8* expression in 535 cancer tissues and 59 adjacent normal tissues. The expression of *CASC8* was significantly higher in NSCLC tissues than in normal tissues adjacent to the tumor (*P* < 0.0001, Fig. 1A). We also assessed *CASC8* expression in cell lines. We used the normal bronchial epithelial cell line HBE and five common NSCLC cell lines for qPCR. The level of *CASC8* was significantly higher in the NSCLC cell lines than in HBE cells (Fig. 1B). We created cells with a stable knockdown of *CASC8*, which showed reduced expression of *CASC8* (Fig. 1C).

### Silencing of *CASC8* inhibits proliferation, migration, and invasion in NSCLC cells

**Figure 2:**
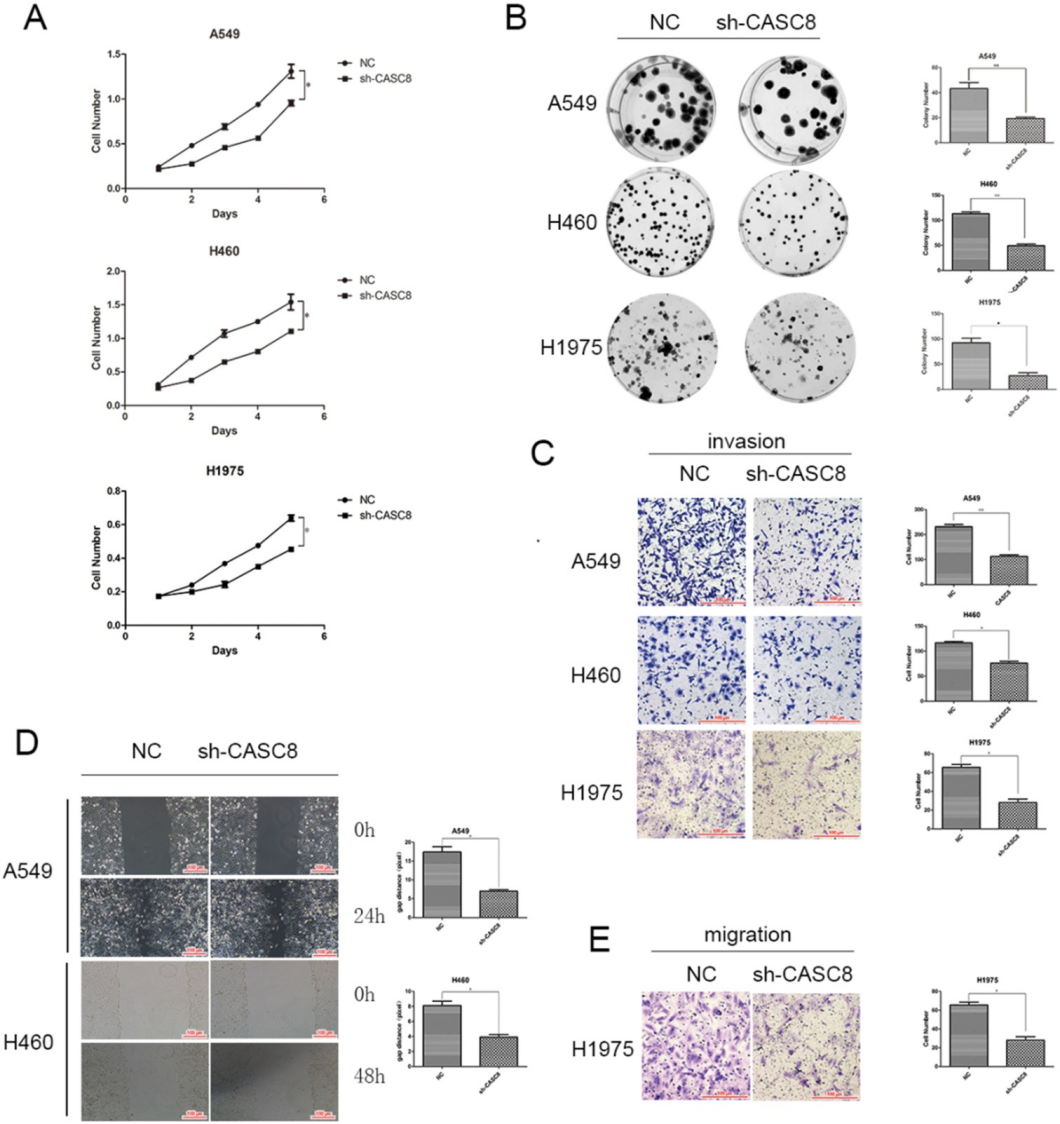
** Silencing of *CASC8* inhibits the proliferation, migration, and invasion of non-small cell lung cancer cells. A.** Silencing of *CASC8* decreased the proliferation of NSCLC cells. Top panel, A549; middle panel, H460; bottom panel, H1975, *P < 0.05. **B.** Colony formation assay to detect the effect of *CASC8* silencing on the colony-forming ability of NSCLC cells. Right panel, quantification, **P < 0.01, ***P < 0.0001. **C.** Silencing of *CASC8* impairs the invasive ability of NSCLC cells (Transwell invasion assay). Left panel, quantification, *P < 0.05, **P < 0.01. **D.** Wound-healing assay using A549 and H460 cells after shCASC8 transfection. The cell migration distance was shorter in shCASC8 cells than in control cells. Right panel, quantification, *P < 0.05. **E.** Transwell migration assay using H1975 cells. Right panel, quantification, *P < 0.05. Abbreviations: CASC8: cancer susceptibility candidate 8; NSCLC: non-small cell lung cancer.

Based on the results of the qPCR and TCGA analyses, we examined the role of *CASC8* in cells. Transfection of shCASC8 in A549, H460, and H1975 cells reduced the expression of *CASC8.* These cells had decreased proliferation (Fig. 2A) and colony-forming ability (Fig. 2B). Invasion was attenuated in cells with *CASC8* knockdown (Fig. 2C), as was cell migration, as determined by the wound-healing assay (Fig. 2D) and the transwell assay without Matrigel (Fig. 2E). These results were statistically significant (*P < 0.05, **P < 0.01). These findings indicate that, as the level of *CASC8* decreases, the migration distance and migration ability of cells decrease. This result corresponds to our previous results, and it can be concluded that decreasing *CASC8* can inhibit vital functions in NSCLC cells.

### *CASC8* regulates genes involved in epithelial-to-mesenchymal transition (EMT) via FOXM1, and FOXM1 expression is associated with poor prognosis in NSCLC

**Figure 3:**
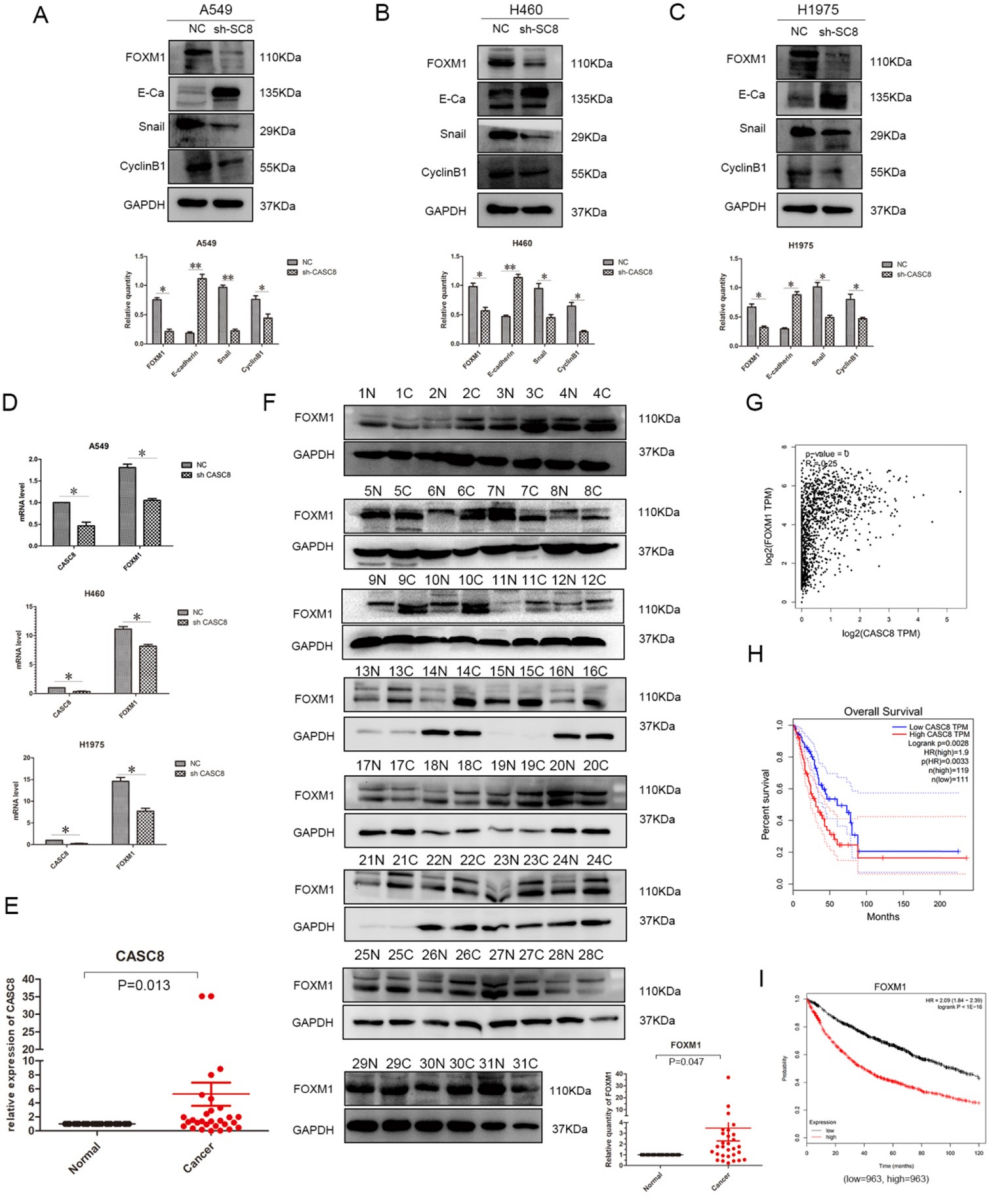
***CASC8* induces EMT via FOXM1, and FOXM1 expression is associated with poor prognosis in non-small cell lung cancer. A.** Changes in expression levels FOXM1, E-cadherin, Snail, and cyclin B1 after CASC8 knockdown in A549 cells. Left panel, quantification, *P < 0.05, **P < 0.01. **B.** As in (A) for H460 cells; **C.** as in (A) for H1975 cells. **D.** The FOXM1 levels decreased after CASC8 silencing. **E.** 31 pairs of clinical tissue samples were collected and qPCR was performed. The expression of CASC8 was higher than in NSCLC tissues in adjacent tissues, *P* < 0.0001. **F.** Western blot analysis was performed using the same tissue as in (Fig. 3E).The expression of FOXM1 was higher than in NSCLC tissues in adjacent normal tissues. Bottom panel, quantification, *P < 0.05, **P < 0.01. **G.** Derived from the GEPIA database, indicating that there is a positive correlation between CASC8 and FOXM1 at the gene level and has statistical significance (P = 0, R = 0.25). **H.** Derived from GEPIA database, high expression of CASC8 is associated with poor patient prognosis in lung adenocarcinoma (*P*=0.0028). **I.** High expression of FOXM1 is associated with poor prognosis of NSCLC, *P* < 0.0001. Abbreviations: CASC8: cancer susceptibility candidate 8; EMT: epithelial-to-mesenchymal transition; FOXM1: Forkhead box M1, NSCLC: non-small cell lung cancer; qPCR: quantitative real-time polymerase chain reaction.

Understanding the effects of *CASC8* on the *in vitro* behavior of NSCLC cells, we further explored its mechanism of action. We assessed the relationship between *CASC8* and FOXM1 using western blotting. When *CASC8* was knocked down, the protein level of FOXM1 decreased, and this was observed in A549 (Fig. 3A), H460 (Fig. 3B), and H1975 (Fig. 3C) cells. *CASC8* silencing increased expression of E-cadherin and decreased expression of Snail. These findings suggest that *CASC8* silencing inhibits EMT. Further, the expression of cyclin B1, a FOXM1 target, was also decreased, which may explain the results of the MTS and colony formation assays. In order to investigate whether *CASC8* regulates FOXM1 at the RNA level, qPCR was performed. The mRNA level of *FOXM1* decreased with *CASC8* knockdown, indicating that *CASC8* regulates *FOXM1* at the transcriptional level. We also selected 31 paired cancer and adjacent tissues for qPCR (Fig. 3E) and western blot (Fig. 3F). Both the levels of *CASC8* and the levels of FOXM1 were higher in the cancer tissues than in the adjacent normal tissues. Through the Gene Expression Profiling Interactive Analysis (GEPIA) database, we obtained the same results as the experimental results, that is, CASC8 and FOXM1 are positively correlated, and the relationship between the two is statistically significant (Fig. 3G, *P* < 0.0001, R=0.25). In addition, we evaluated the relationship between CASC8 expression and FOXM1 expression and patient prognosis using the GEPIA database and the Kaplan-Meier plotter database. *CASC8* expression (Fig. 3H, *P* =0.0028) and FOXM1 expression (Fig. 3I, *P* < 0.0001) were associated with poor prognosis in NSCLC.

### Silencing of CASC8 promotes sensitivity to osimertinib in NSCLC cells

**Figure 4:**
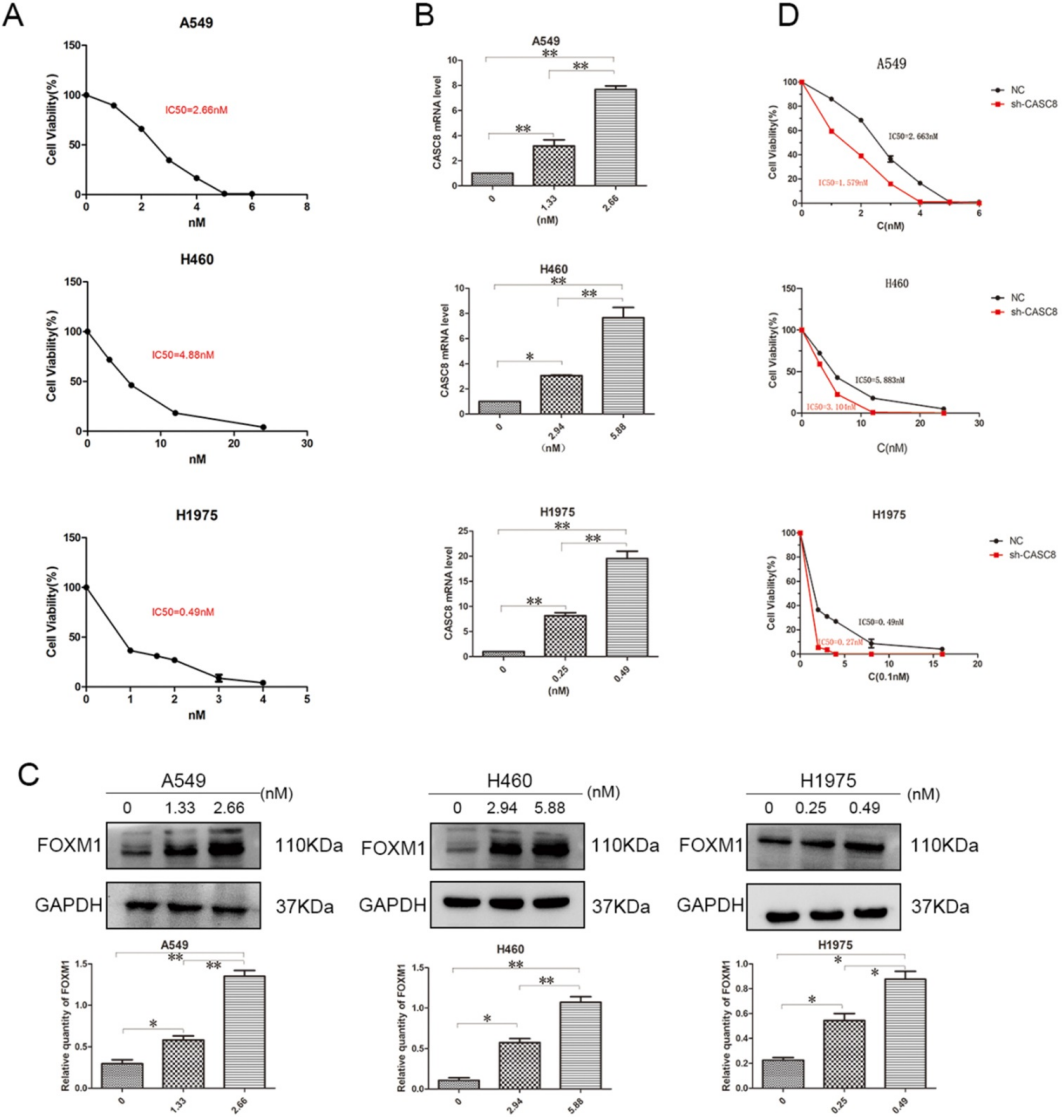
** Silencing of *CASC8* promotes sensitivity to osimertinib in non-small cell lung cancer cells. A.** The half-lethal dose (IC50) of osimertinib was determined in A549, H460, and H1975 cells using an appropriate concentration gradient. After 48 hours of culture, the IC50s of A549, H460, and H1975 cells were 2.663 nM, 5.883 nM, and 0.49 nM, respectively. **B.** Concentrations of osimertinib corresponding to the appropriate IC50 and half the IC50 were added to the three cell lines. Cells were cultured for 48 hours and RNA was extracted for qPCR. The change in *CASC8* expression was osimertinib-dependent. **C.** Cells were cultured for 48 hours at different drug concentrations and then subjected to western blot analysis. The change in the FOXM1 levels was osimertinib-dependent. Bottom panel, quantification, *P < 0.05, **P < 0.01. **D.** The IC50s of shCASC8 A549, H460, and H1975 cells were significantly lower than those of their corresponding NC cells, *P < 0.05, **P < 0.01. Abbreviations: CASC8: cancer susceptibility candidate 8; FOXM1: Forkhead box M1, qPCR: quantitative real-time polymerase chain reaction; NC: negative control.

Targeted drugs are very important in the treatment of NSCLC, but resistance is an issue. Therefore, we examined the ability of *CASC8* to act as a mechanism of resistance to the targeted drug osimertinib. First, to test the IC50 of osimertinib in A549, H460, and H1975 cells, a concentration gradient of osimertinib was added to the cells. After 48 hours, the IC50s of osimertinib in A549, H460, and H1975 cells were 2.663 nM, 5.838 nM, and 0.49 nM, respectively (Fig. 4A). Next, concentrations of osimertinib corresponding to the individual IC50s and half the concentration of the IC50s were added, and the *CASC8* and FOXM1 levels were assessed. In all three cell lines, the level of *CASC8* increased with increasing drug concentration, which proved that the increase in *CASC8* was osimertinib-dependent (Fig. 4B). Similar results were obtained in western blot experiments. FOXM1 was also increased in an osimertinib-dependent manner (Fig. 4C). These results indicate that both *CASC8* and FOXM1 have a role in resistance to osimertinib. After silencing *CASC8*, the IC50s of A549, H460, and H1975 cells decreased to 1.579 nM, 3.104 nM, and 0.27 nM, respectively (Fig. 4D). A colony formation experiment was conducted and it was found that when adding the IC50 concentration of osimertinib to the A549 cells, H460 cells and H1975 cells, the cells with lower *CASC8* expression had the worst colony formation ability (Supplementary A). These experiments confirm our hypothesis that silencing *CASC8* can promote sensitivity to osimertinib in NSCLC cells, which provides new insights for its clinical application.

## Discussion

NSCLC has a huge burden on human health and life worldwide, and the treatment of NSCLC has caused enormous economic burdens for individuals and society. Research and treatment into the development of NSCLC is very important. In this study, the role of the lncRNA *CASC8* in NSCLC was identified.

First, we showed that the expression of *CASC8* was significantly higher in tumors than in adjacent tissues using the TCGA database, which provides a rationale for the study of *CASC8*. The A549 and H460 NSCLC cell lines, which have relatively high levels of *CASC8,* and the H1975 NSCLC cell line, which harbors an EGFR T790M mutation, were selected as experimental models for this study, and a series of *in vitro* experiments were performed.

*In vitro* behavioral experiments, we learned that silencing of *CASC8* inhibits the proliferation, migration, and invasion of NSCLC cells. In addition, the expression of cyclin B1, which is associated with the G2/M transition, decreased with *CASC8* knockdown, which corresponds to the results of the MTS assay and the colony formation assay. We were intrigued to find that FOXM1 also decreased with *CASC8* knockdown. FOXM1 is a transcription factor that is required for cell proliferation, promotes tumor development, and is an oncogene 19. FOXM1 promotes proliferation in NSCLC cells, and it can promote the transition from G2 to M phase via transcriptional regulation of cyclin B1 20-22. This result further underscored the role of *CASC8* in NSCLC cells. However, how *CASC8* affects FOXM1 is still unknown. The miRTarBase indicates that *CASC8* can interact with miR-671-5P, and miR-761-5P can inhibit the transcription of *FOXM1* 23. Therefore, we hypothesize that *CASC8* can act as a sponge of miR-671-5P. However, the exact role of *CASC8* and miR-671-5P was not explored in this study, and this should be further verified. But through the GEPIA database and our experiments, we can prove that there is a positive regulatory relationship between CASC8 and FOXM1, and the relationship between the two is statistically significant. At the same time, the high expression of both is related to the poor prognosis of patients (from GEPIA and Kaplan Meier-Plotter).

FOXM1 is also essential for pulmonary fibrosis and EMT 24. FOXM1 regulates the expression of E-cadherin 20,25, and E-cadherin downregulation is a hallmark of EMT. When the E-box sequence in the E-cadherin promoter is inhibited, Snail can bind to the E-box sequence, resulting in decreased expression of E-cadherin 26-28. Snail plays a vital role in EMT as a procedural switch 29. When E-cadherin expression is reduced, tumor cells undergo migration and invasion 30. We observed several potential EMT-related changes with *CASC8* knockdown, such as an increase in E-cadherin and a decrease in Snail. This may explain the decreased migration and invasion in NSCLC cell lines after *CASC8* silencing.

Only a portion of early lung cancer can be cured by surgical resection. Further, more than 60% of patients with lung cancer are diagnosed with locally advanced lung cancer or have lymph node metastasis (stage III or IV), and surgery is not the primary treatment option 5. *EGFR* is a commonly mutated gene in NSCLC. Although some therapeutic targets have been identified, patients inevitably develop resistance, limiting the utility of targeted therapies. Therefore, identifying mechanisms of resistance is vital for improving patient survival.

EGFR-targeted treatment is widely used in the clinic for patients with lung cancer. Early tyrosine kinase inhibitors (TKIs) such as gefitinib and afatinib have good effects in the initial stage of treatment, but the effect cannot be extended beyond 10-14 months, and patients inevitably develop resistance 31-34. Interestingly, FOXM1 also plays a role in NSCLC resistance to gefitinib, 22 although the most common cause of drug resistance is the T790M mutation 35. Osimertinib is a third-generation TKI that has been proved to overcome the EGFR T790M mutation 36 and shows better clinical efficacy that gefitinib and erlotinib 37. Although tumors are more sensitive to later-generation EGFR-TKIs, there is still a drug resistance phenomenon 38,39. In this study, we demonstrated that silencing of *CASC8* can sensitize NSCLC cells to osimertinib, identifying a potential mechanism of osimertinib resistance. However, whether the silencing of *CASC8* promotes sensitivity to osimertinib *in vivo* has not been verified and needs to be confirmed.

## Conclusions

In summary, the lncRNA *CASC8* promotes the proliferation, migration, and invasion of NSCLC cells. *CASC8* also sensitizes NSCLC cells to osimertinib. However, whether *CASC8* can be used as a standard for the diagnosis of NSCLC still requires further experimental verification.

## Supplementary Material

Supplementary figure.

## Abbreviations

NSCLC: non-small cell lung cancer
EGFR: epidermal growth factor receptor
lncRNA: long noncoding RNA
miRNA: microRNA
CASC8: cancer susceptibility candidate 8
NC: negative control
FOXM1: Forkhead box M1
qPCR: quantitative real-time polymerase chain reaction
EMT: epithelial-to-mesenchymal transition
MTS: 3-(4,5-dimethylthiazol-2-yl)-5-(3-carboxymethoxyphenyl)-2-(4-sulfophenyl)-2H-tetrazole
TCGA: The Cancer Genome Atlas
TKI: tyrosine kinase inhibitor
shRNA-GFP: scramble small hairpin RNA-green fluorescent protein
FBS: fetal bovine serum
HBE: human bronchial epithelial
DMEM: modified eagle medium
RPMI: Roswell Park Memorial Institute
TTBS: Tween Tris buffered saline
GAPDH: Glyceraldehyde 3-phosphate dehydrogenase
RNA: Ribonucleic acid
cDNA: complementary deoxynucleic acid
GEPIA: Gene Expression Profiling Interactive Analysis
