# Supplementary material for: Silencing of CASC8 inhibits non-small cell lung cancer cells function and promotes sensitivity to osimertinib via FOXM1
Source: J Cancer. 2021 Jan 1;12(2):387–96. doi: 10.7150/jca.47863 (PMC7739000; doi:10.7150/jca.47863)
Supplement: Supplementary file 1 — Supplementary figure. [file jcav12p0387s1.pdf]

A

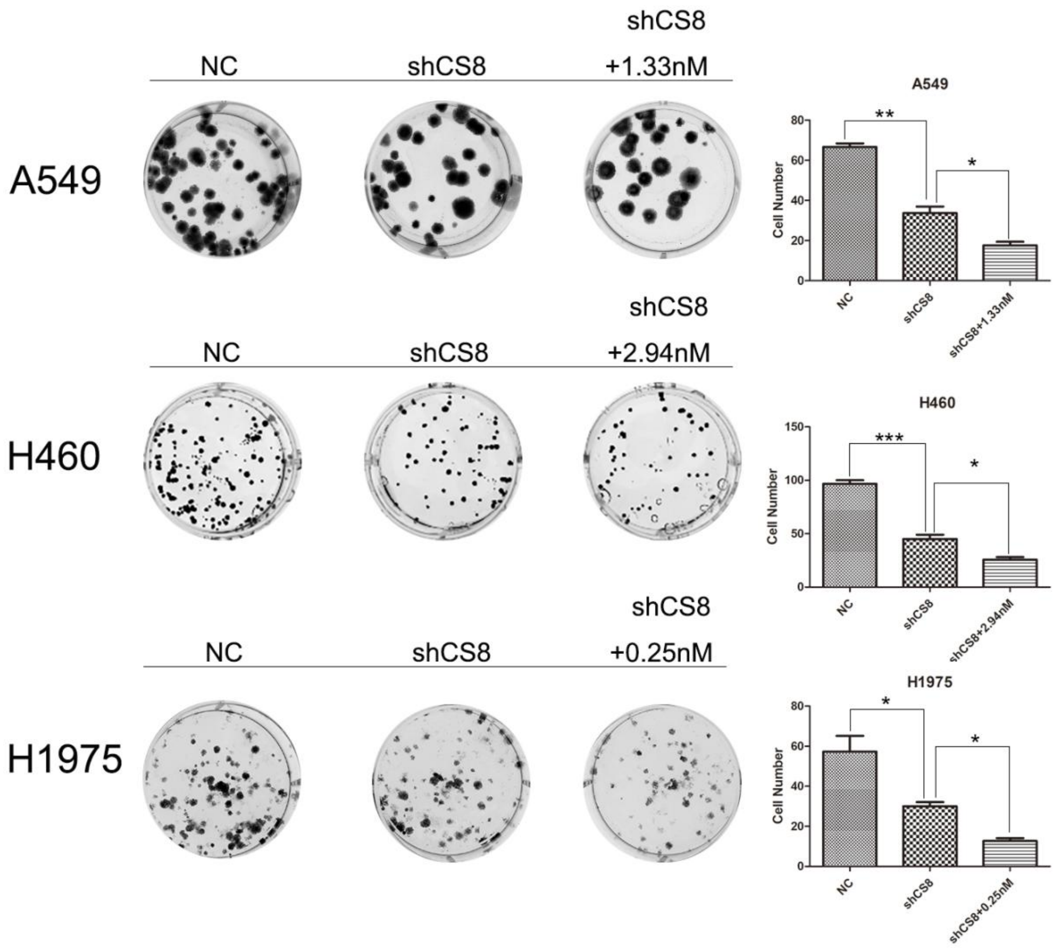

Supplementary

A. The colony formation experiments of A549 cells, H460 cells and H1975 cells, from left to right, were the negative control group (NC), the corresponding IC50 concentration of osimertinib experimental group was added, and the experimental group that added IC50 concentration of osimertinib after reducing the expression of CASC8 (*P < 0.05, **P < 0.01.***P<0.001).
